# Thraustochytrid hosts for expression of proteins relevant to SARS-CoV-2 intervention

**DOI:** 10.1371/journal.pone.0283592

**Published:** 2023-04-12

**Authors:** Jeremy Dahmen, Arjan Vermeulen, Sophie Payne, Casey Lippmeier

**Affiliations:** Conagen, Inc, Bedford, MA, United States of America; Arizona State University, UNITED STATES

## Abstract

The emergence of COVID-19 as a global pandemic had sharply illustrated the limitations of research and development pipelines and scaled manufacturing. Although existing vaccines were created in record time, global deployment remains limited by regional production scales. Similarly, the most effective treatments for infected COVID-19 patients are also constrained by production scales as well as by the cost of production and thus expense per treatment. The need to produce these interventions more cost-effectively, at larger scales, in less time while retaining high quality is paramount. The Conamax^TM^ platform is based on a Thraustochytrid–an order of microorganisms well established in industry for world-scale production of omega-3 fatty acids by fermentation. Thraustochytrids, and the species *Aurantiochytrium acetophilum* in particular, possess a number of innate qualities which make it ideal for production of monoclonal antibodies and other biotherapeutic proteins. In this study, the Conamax system was used to produce several targets which may be relevant as interventions in the fight against COVID-19; an anti-SARS-CoV-2 antibody (CR3022), tocilizumab, and the ACE2 receptor. Our system was capable of producing all of these targets and each was assayed *in vitro* for an activity which confirmed proper structural folding. Purified CR3022 antibody produced from Conamax was capable of reducing the cytopathic effect of SARS-CoV-2. Conamax-derived tocilizumab was shown to bind to its target IL6R. Both the full-length and soluble versions of ACE2 protein produced in the Conamax platform exhibited ACE2-specific proteolytic activity. These data indicate that the Conamax platform has great potential in the production of therapeutic agents.

## Introduction

The Coronavirus disease 2019 (COVID-19) is caused by the severe acute respiratory syndrome coronavirus 2 (SARS-CoV-2) and has resulted in over 5.3 million deaths globally [1]. Several prevention and treatment options have now been developed and deployed globally. These and other potential interventions include 1) anti-SARS-CoV-2 CR3022 monoclonal antibody (mAb) for spike protein detection or as a therapeutic candidate, 2) tocilizumab, an anti-inflammatory mAb approved for use in mitigating the COVID-associated “cytokine storm” and 3) both full-length and soluble angiotensin-converting enzyme 2 (ACE2) signal receptor as a potential therapeutic candidate [2–5].

Antibodies against the spike protein can serve as candidates for therapeutic treatment. One such antibody ostensibly capable of cross-neutralizing SARS-CoV-2 is CR3022, originally obtained from a convalescent SARS-CoV infected patient [6]. This antibody binds to the receptor binding domain (RBD) of the SARS-CoV-2 spike protein and therefore has been considered as a potential therapeutic mAb for treating COVID-19 [7].

COVID-19 infection can lead to a release of a large amount of pro-inflammatory cytokines including interleukin 6 (IL-6) in an event known as the “cytokine storm” [8, 9]. The host immune response to the SARS-CoV-2 virus becomes hyperactive, resulting in an excessive inflammatory reaction. Several studies analyzing cytokine profiles from COVID-19 patients suggested that the cytokine storm correlated directly with lung injury, multi-organ failure, and unfavorable prognosis of severe COVID-19. Tocilizumab, an IL-6 receptor-binding antibody, may reduce this cytokine storm and thus have an important role in the treatment of patients with COVID-19 [8].

The ACE2 protein is the cell-surface ligand of the SARS-CoV-2 spike protein, comprised of 805 amino acid residues containing a single transmembrane domain. During COVID-19 infection, recombinant, soluble ACE2 can serve as a decoy competing for binding sites on the spike protein therefore reducing infectious SARS-CoV-2 titers. One recent study [10] used human recombinant soluble ACE2 to successfully treat an individual with a severe SARS-CoV-2 infection.

In the present study, we demonstrated expression of a number of potential targets for development of therapeutic and vaccine targets for COVID-19 using the Conamax platform.

The Conamax platform is based on a wild isolate of the order Thraustochytrida, *Aurantiochytrium acetophilum*. The use of this platform for the production of the mAb trastuzumab was previously described [11]. In that study, the authors compared structural qualities of their trastuzumab to the same antibody as produced by Chinese hamster ovary cells (glycostructures, aggregates, total intact mass), tested the efficacy of a trastuzumab antibody drug conjugate (ADC) in a mouse xenograft model of Her2+ gastric cancer, and demonstrated that the Conamax-produced antibody was able to prevent tumor growth as effectively as authentic trastuzumab in this ADC format. The platform has since been used to produce additional mAbs, and in this study we demonstrate how the platform may also serve as a valuable tool to rapidly produce other glycosylated proteins for COVID-19 treatment and detection.

## Materials and methods

### Strains and growth conditions

*A*. *acetophilum* was isolated by Synthetic Genomics Incorporated and subsequently acquired by Conagen. *A*. *acetophilum* cells were typically grown at 30° C; 180 rpm in FM02 medium [12].

### Vector constructs, and transformation

The heavy and light chain sequences for SARS-CoV-2 CR0322 antibody were obtained from GenBank under the accession numbers DQ168569 and DQ168570, respectively. The tocilizumab heavy and light chain sequences were obtained from literature [13]. The full-length and soluble portions of ACE2 were each identified from GenBank accession number BAB40370. The CR3022 antibody, tocilizumab, and ACE2 sequences were each codon optimized for *A*. *acetophilum* using a codon table (S1 Table) generated from the genome sequence previously constructed in our lab.

A previously identified secretion signal from *Batrachochytrium dendrobatidis*, 579SS, MPFNRLSLPCLLLALIASLFIHAAQAG, was fused to the N-terminal end of the genes encoding CR3022 antibody, and tocilizumab to enable improved protein secretion [12]. Golden Gate assembly [14] using the *Bsa*I restriction site to clone each of the COVID19-target sequences including the CR3022 antibody, tocilizumab, full-length and soluble ACE2 genes with a native promoter, TubB or Tef, a native terminator, Eno2, and a pET backbone carrying ampicillin resistance for *E*. *coli* propagation and paromomycin resistance, was used for preparation of genetic constructs for thraustochytrid transformations.

*A*. *acetophilum* (for expression of CR3022, tocilizumab, or ACE2) was cultured to log-phase for transformation in FM2 medium and electroporated with approximately 2 μg of *Sbf*I-linearized plasmid at 700 V, 200 Ω, 25 μF. Cells recovered for 16 hours in GY medium and were plated onto solid FM2 medium containing 1.5 g/L paromomycin [12]. Antibiotic resistance was confirmed by transferring colonies to a fresh selective plate. Primary transformants were further verified by colony-polymerase chain reactions directed to the gene of interest. These methods resulted in the final strains listed below in Table 1.

**Table 1:** Strains used to confirm production of monoclonal antibodies and proteins.

Strain Designation	Product
STR176	None (wt)
SCX00040	CR3022
sCX00023	Tocilizumab
sCX00037	Full-length ACE2
sCX00148/sCX00149	Truncated, soluble ACE2s

### SDS-PAGE and western blotting

Samples were prepared with SDS containing sample buffer (G Biosciences, St. Louis, MO), and heated at 70°C for 10 minutes. For reducing conditions, dithiothreitol was added to a final concentration of 50 mM. Proteins were separated on 10–20% Tris-Glycine or 4–12% Bis-Tris gels (WedgeWell and Bolt respectively, Invitrogen, Carlsbad, CA). For western blotting, proteins were transferred to a nitrocellulose membrane using the Trans-Blot semi-dry transfer apparatus (Bio-Rad, Hercules, CA). Membranes were blocked in 5% (w/v) non-fat dry milk in Tris-buffered saline with 0.1% (v/v) Tween-20 (TBST) for 1 hour at room temperature or overnight at 4°C. Blocked membranes were then probed with antibodies against the protein of interest. To detect ACE2, a rabbit polyclonal antibody against the protein (Sino Biological, Wayne, PA, cat. no. 10108-T60) was used. As secondary antibody, goat anti-rabbit IgG conjugated with alkaline phosphatase was used (Southern Biotech, Birmingham, AL, cat. no. 4030–04). To detect CR3022 or tocilizumab, anti-human IgG gamma-AP (Rockland Immunochemicals, Limerick, PA, cat.no. 609–35663) or anti-human IgG kappa-AP (Novus Biologicals, Centennial, CO, cat.no. NB7464) were used. Finally, membranes were then stained with NBT/BCIP (Fisher Scientific).

Capillary electrophoresis-PAGE (CE-PAGE) was done using a LabChip GXII Touch HT (Perkin-Elmer, Billerica, MA) with the Protein Express kit (Perkin-Elmer, Billerica, MA). The manufacturer’s procedure was followed, with the exception that samples were heated to 70°C for 10 minutes.

### Purification of antibodies from culture supernatants

Secreted antibodies were purified by Protein A chromatography. Harvested culture supernatants were first flocculated with polyDADMAC (EMD Millipore, Burlington, MA), followed by centrifugation for 20 minutes at 15,000 *g*. The clarified supernatants were filtered and applied to 5 mL Praesto Jetted A50 resin (Purolite, King of Prussia, PA) in a XK26/20 column (Cytiva, Marlborough, MA) using an AKTA explorer FPLC. The column was first equilibrated with phosphate-buffered saline (PBS) (Boston BioProducts, Ashland, MA). Prior to elution with 0.1 M acetic acid, the column was washed with 10 column volumes PBS. The pH of the eluted fractions was adjusted by adding 1 M Tris-HCl (pH 9).

### Virus neutralization assay

Virus neutralization assays were performed by the Institute for Antiviral Research (Utah State University). Purified CR3022 was diluted in Minimal Essential Medium (MEM) with 2% fetal bovine serum (FBS) (Sigma-Aldrich) and 50 μg/mL gentamycin (Caisson Laboratories, Smithfield, UT) to 200 μg/mL. This was then diluted in a 2-fold series. The diluted antibodies were mixed with SARS-CoV-2 (strain USA_WA1/2020, diluted to lowest MOI that would yield >80% cytopathic effect) and incubated for 1 hour at 37°C. After incubation, the mixture was transferred to a 96-well plate with 80–100% confluent Vero 76 cells. For each dilution, three wells were infected with virus and 2 wells remained uninfected as toxicity controls. Infected/untreated and uninfected/untreated controls were included. The protease inhibitor M128533 (Epicept Corp, San Diego, CA) was tested as a positive control.

After 5 days of incubation–at which point most untreated, infected cells reached maximum cytopathic effect (CPE) [15]–the plates were stained with Neutral Red dye for approximately 2 hours. Cells were washed, and incorporated dye extracted with 50:50 Sorensen citrate buffer/ethanol and the absorbance was measured at 540 nm [16]. These optical densities were converted to percent of cell control and normalized to the virus control. Regression analysis was performed to estimate the concentration of CR3022 needed to inhibit cell death to 50% (EC_50_).

Viral yield reduction was determined by collecting supernatant fluid at day 3 post-infection. Viral titer was determined using a standard endpoint dilution CCID_50_ assay [15]. Titers were calculated using the Reed-Muench equation [17]. Regression analysis was used to calculate the concentration of CR3022 needed to reduce the virus yield by 90% (CC90).

### IL6R-binding assay

Binding of tocilizumab to IL6R was determined by ELISA. An ELISA plate (Nunc-Immuno MaxiSorp, Thermo Fisher Scientific, Waltham, MA) was coated with IL6R (Genscipt, Piscataway, NJ). IL6R was diluted in carbonate/bicarbonate buffer (pH 9.4) to 10 μg/mL, and then diluted in a 2-fold series. Each well was coated with 100 μL diluted IL6R. After overnight incubation at 4°C, plates were blocked with 5% non-fat dry milk in PBS for 1 hour at room temperature, with shaking at 400 rpm. Plates were washed with TBST.

Conamax-produced tocilizumab and a commercial tocilizumab preparation (Novus Biologicals, Centennial, CO, NBP2-75192) were diluted to 2 μg/mL. As a negative control, Conamax-produced trastuzumab was included [11]. Per well, 100 μL of diluted antibody was added and incubated at room temperature for 2 hours (with shaking at 400 rpm). After incubation, plates were washed and probed with anti-human IgG Fc conjugated with HRP (Southern Biotech, Birmingham, AL, cat.no. 2081–05). Plates were washed and 100 μl 3,3’,5,5’-tetramethylbenzidine (Bio-Rad, Hercules, CA) was added to each well. Reactions were stopped with stop solution (Abcam, Waltham, MA) and absorbance measured at 450 nm with a SpectraMax i3 plate reader (Molecular Devices, San Jose, CA).

### Partial purification and activity of ACE2

ACE2 and ACE2 lacking the membrane-binding domain (ACE2s) were produced by growing strains sCX00037 and sCX00148 respectively, in FM02 medium. Culture supernatants were collected by centrifugation at 3000 x *g* after overnight incubation at 30°C. The harvested culture supernatants were then dialyzed overnight against 2 liters of 25 mM Tris-HCl (pH 8). Dialyzed supernatants were then loaded to a Q Sepharose column (Cytiva, Marlborough, MA) equilibrated with 25 mM Tris-HCl (pH 8). Proteins were eluted with a linear gradient to 1 M NaCl. Elution fractions were tested for presence of ACE2(s) by western blot and ELISA (Human ACE-2 DuoSet ELISA kit, R&D Systems, Minneapolis, MN). Fractions with the highest concentration ACE2(s) were then tested for ACE2 activity using a commercial activity assay (BioVision, Milipitas, CA). Non-specific protease activity was determined by adding ACE2 inhibitor to samples before testing their activities. The manufacturer’s instructions were followed with the exception that the provided positive control was diluted to similar activity levels.

## Results

### CR3022 production and virus neutralization activity

**Fig 1:**
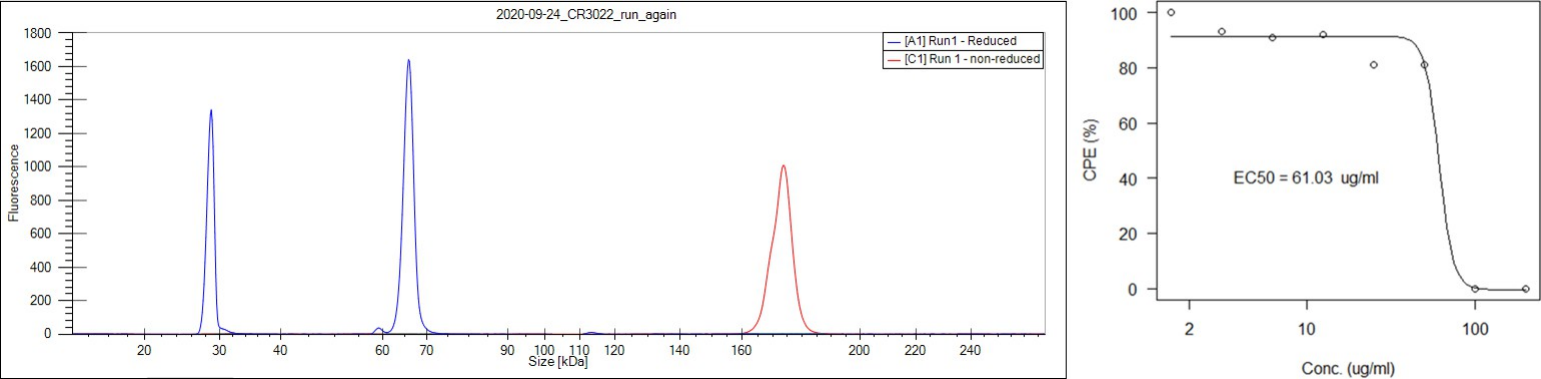
a. CE-PAGE profile of purified CR3022. The Conamax-produced antibody was purified to an apparent 100% purity by a single Protein A chromatography step. Red curve: non-reduced sample, blue curve: reduced, showing heavy and light chains. b. Antibody CR3022 produced by Conamax was able to inhibit induced cytopathy by SARS-CoV-2 in Vero 76 cells. The EC_50_ was 61 μg/ml in this assay. Data shown here are averages of three observations per concentration of CR3022.

We generated an *A*. *acetophilum* strain carrying genes encoding single copies of both heavy and light chains of CR3022 (sCX00040). The strain sCX00040 secreted assembled CR3022 into the culture supernatant. A fed-batch fermenter culture achieved supernatant titers around 20 mg/L. CR3022 was purified to 100% purity (as determined by CE-PAGE) with a single Protein A purification (Fig 1A). The resulting preparation was effective in reducing the CPE of SARS-CoV-2 in Vero 76 cell cultures (Fig 1B). The EC_50_ was 61 μg/mL, which is in line with previously reported numbers (15).

### Tocilizumab production and IL6R binding

**Fig 2:**
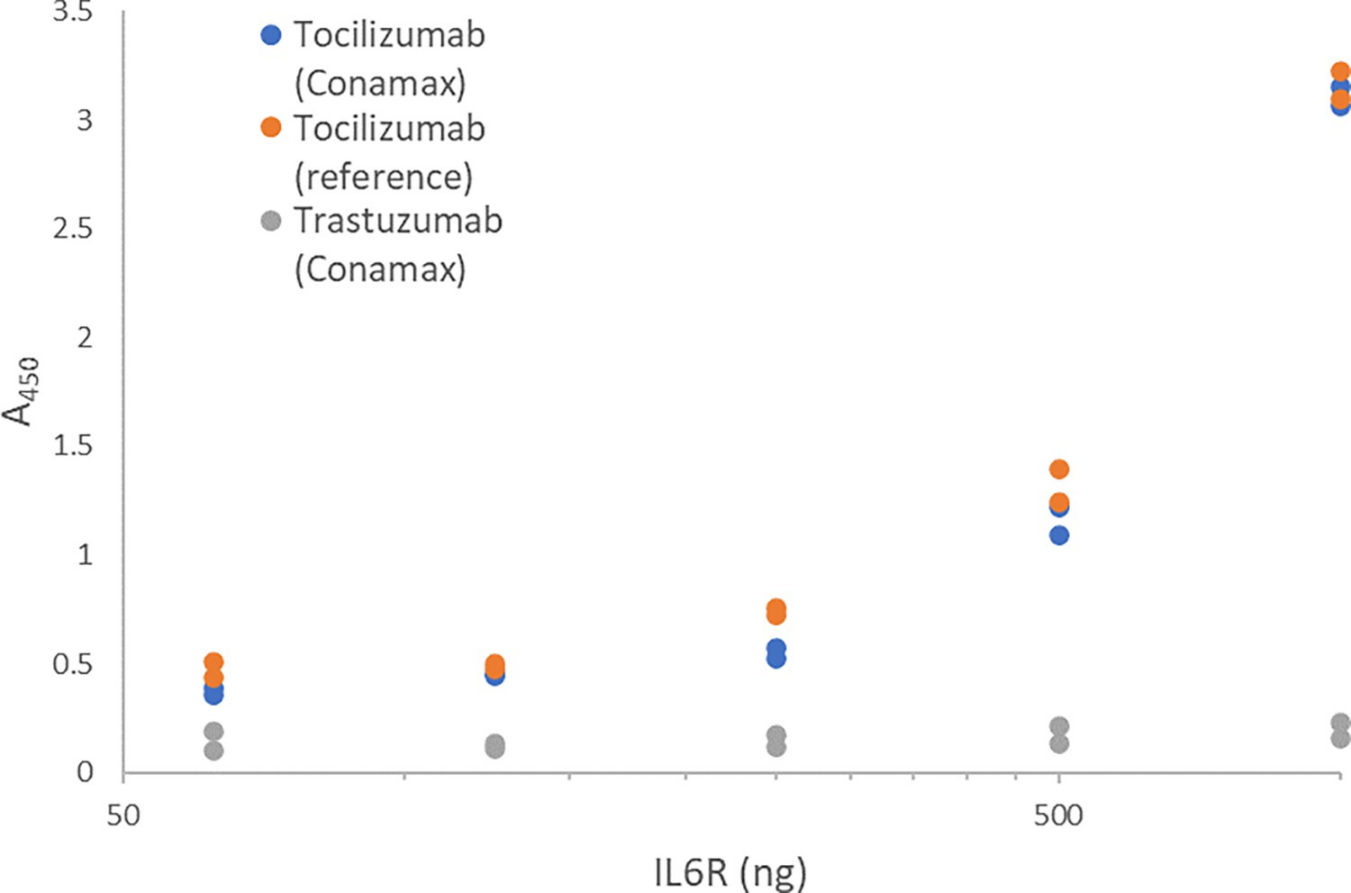
Tocilizumab produced with Conamax binds IL6R with a similar affinity as a reference sample of tocilizumab. Binding was tested using an ELISA plate coated with IL6R. As control, trastuzumab produced by Conamax was used. As expected, this negative control antibody did not show any binding.

An *A*. *acetophilum* strain carrying single copies of genes encoding the heavy and light chains of tocilizumab was generated. The strain sCX00023 readily secreted tocilizumab to the culture supernatant. Overnight shake flask cultures typically reached titers around 1–1.5 mg/L. Tocilizumab was purified from the clarified supernatants by a single Protein A chromatography step. We confirmed binding of the produced tocilizumab to its target, IL6R, by ELISA. The affinity was similar to that of a commercially available sample of tocilizumab (Fig 2).

### ACE2 production and activity

**Fig 3:**
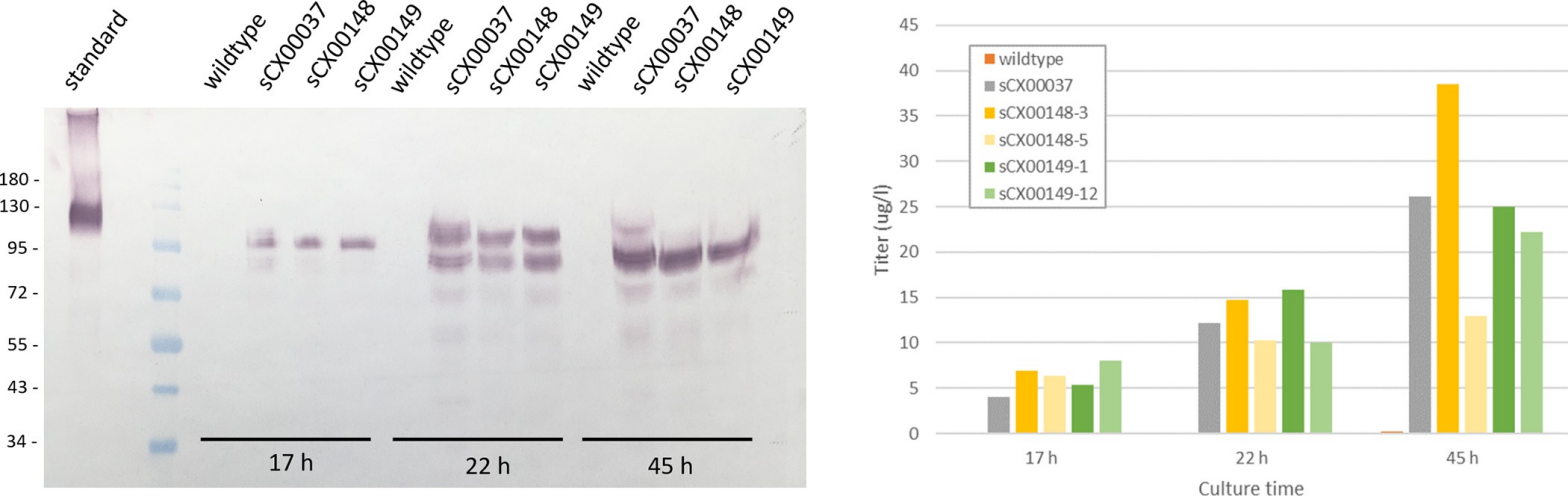
a. Expression and secretion of ACE2. Strain sCX00037 produced full-length ACE2, while strains sCX00148 produced a truncated form of ACE2. After prolonged incubation, some clipping of the produced ACE2 is noticeable. The specificity of the detection antibody was confirmed by the absence of bands in the wildtype samples. b. Titers of produced ACE2. Titers were determined using a sandwich ELISA. A steady increase in titers was seen, but as the western blot shows, this came with an increase in clipped ACE2.

We generated *A*. *acetophilum* strains that were able to produce ACE2 (strain sCX00037) and a truncated form of ACE2 lacking the membrane-binding domain (ACE2s; strains sCX00148). Secretion of ACE2 and ACE2s was confirmed by western blot and a sandwich ELISA specific for ACE2 (Fig 3). Titers of secreted ACE2 from shake flask cultures were around 5 μg/L after overnight incubation. Titers increased to 25–40 μg/L after incubation for 45 hours. However, western blots showed accumulation of what looked like truncated ACE2(s) at 22 hours (Fig 3A). Although the Western Blot is not ideal for resolving the size difference between the truncated and full-length forms, the band sizes do appear slightly larger than expected.

**Fig 4:**
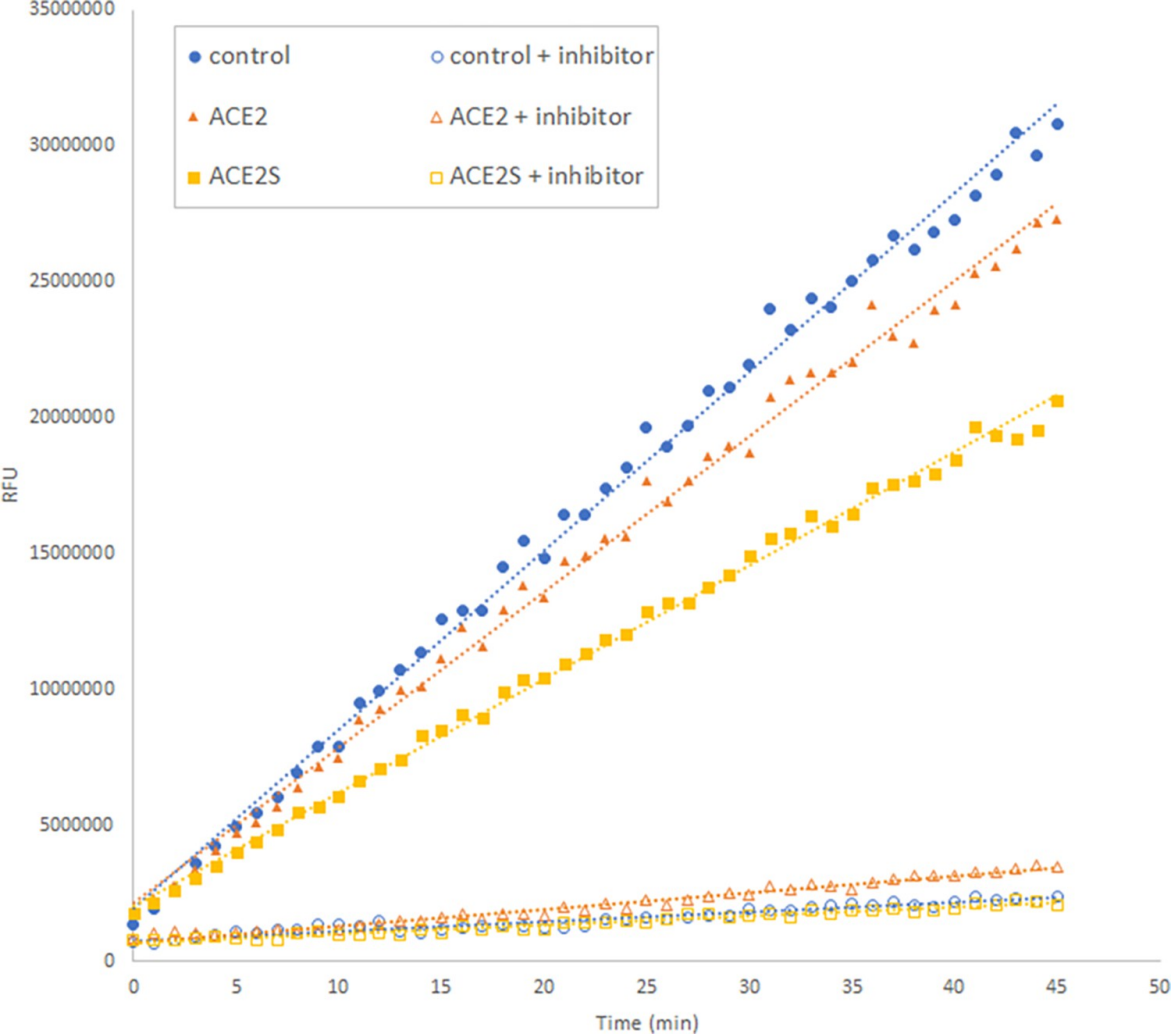
Enriched culture supernatant samples show ACE2 activity. Proteolytic activity was determined using a fluorescently labeled probe. Non-specific proteolytic activity was determined by adding an ACE2 inhibitor. As a control, ACE2 supplied with the activity assay was used.

We enriched the culture supernatants by anion exchange chromatography. ACE2 and ACE2s eluted early at low ionic strength. The fractions with the highest concentration of ACE2 were then tested for proteolytic activity (Fig 4). To confirm that this activity was indeed ACE2-specific, we also included control reactions where an ACE2 inhibitor was added. These reactions indeed showed very little activity in comparison to the reactions without inhibitor. This confirmed that the produced ACE2 was active.

## Discussion

As COVID-19 cases continue to rise along with viral mutations which have led to increased transmissibility, the need for additional world-scale production of antibodies and therapeutic agents will remain [18]. It is vital to identify new and emerging systems that produce vaccines and therapeutics faster and cheaper.

An emerging system for the production and secretion of monoclonal antibodies and biotherapeutic agents was used to express and analyze several COVID-related targets. A previous study on the Conamax platform focused on an in-depth analysis of Conamax-derived trastuzumab and comparisons with trastuzumab produced from Chinese hamster ovary (CHO) cell cultures [11]. In the present study, we produced a range of both monoclonal antibodies as well as full length and soluble proteins all related to COVID-19 mitigations to gain an improved understanding of the capabilities of the Conamax platform. The three targets we chose included CR3022 antibody, tocilizumab, and both full-length and soluble ACE2 protein.

Since being identified as the primary receptor for SARS-CoV-2, human ACE2 has been a targeted treatment option. At 802 amino acids, human ACE2 is a very large protein containing a single transmembrane domain. The soluble portion of ACE2 has been used previously to neutralize SARS-CoV-2 by serving as a decoy which competes for receptor sites with wild type ACE2. We were able to successfully express both the full-length ACE2 and soluble portion of ACE2 in *A*. *acetophilum*. ACE2 also has a proteolytic domain, thus it was important to determine if our thraustochytrid-derived ACE2 has activity. Based on these assays, we confirmed that both full-length and soluble versions of ACE2 show ACE2-specific activity, demonstrating that the Conamax system produces both forms of this protein in a structurally relevant context. We would expect the full-length protein to have better activity than the soluble portion of ACE2 because of the way it is presumably expressed in Conamax (embedded in extracellular exosomes). Further, we also expect full-length, transgenic ACE2 to be closer to its native configuration than the truncated form and therefore retain higher activity [19].

We were able to successfully express CR3022 antibody using the Conamax host, *A*. *acetophilum*. In our study, purified CR3022 antibody demonstrated an ability to reduce the CPE of SARS-CoV-2 comparable to values reported for CR03022 produced by other systems [15, 20]. Although CR3022 does not serve as a neutralizing antibody, the comparable moderate reduction in infectivity observed with the Conamax version of the antibody indicates that the binding sites of Conamax-CR3022 should be structurally similar to CR3022 produced in other systems [20].

COVID-19 can lead to inappropriately increased production of cytokines such as IL6. This can in turn lead to increased production of a variety of pro-inflammatory cytokines downstream of the IL6 binding event to IL6-R (ie: the “cytokine storm”). Another potential COVID-19 therapeutic target, tocilizumab, has the ability to bind IL-6R, therefore blocking the downstream effects of IL6 over-induction. We have expressed tocilizumab using the Conamax host *A*. *acetophilum*. This Conamax-derived tocilizumab is also capable of binding IL6R similarly to that of commercially available tocilizumab. The capability of microbes to produce fully functional antibodies is not trivial, requiring proper assembly and folding at both secondary and tertiary structural levels, proper transit through the secretory pathway, and correct glycosylation [21–23]. With the antibodies CR3022 and tocilizumab, we have presented two more examples of functional *A*. *acetophilum*-derived monoclonal antibodies.

The studies illustrated in this work indicate that the Conamax platform can be used to express several antibodies and therapeutic agents for pharmaceutical applications. The system produces functional monoclonal antibodies including CR3022 and tocilizumab and active proteins, as is the case with ACE2. This initial study was primarily used as a proof of concept to determine if we could produce COVID-19 monoclonal antibodies and therapeutic agents in our Conamax platform. Although our initial unoptimized strains result in low titers when cultivated in shake flask or benchtop fermenters (within the range of 1–20 mg/L), the host *A*. *acetophilum* is highly amenable to microbial engineering as demonstrated in our prior work where subsequent rounds of ectopic transformation and adapted laboratory evolution led to increased productivities up to 1 g/L/d of target mAb [11, 12]. Ongoing development of our fermentation processes are expected to increase these titers and productivities even further. Future work will also focus on improvements to our downstream process and other parameters necessary for production of pharmaceutically-relevant proteins at reduced cost.

By previous and present studies, the Conamax platform has great potential in producing various recombinant proteins as pharmaceuticals. The supernatant of Conamax is simpler than traditional CHO cell antibody-production systems, reducing downstream processing time and cost considerably. Coupled with its capability for large scale culture, Conamax has substantial value as a better, low-cost, and faster-to-market biopharmaceutical production platform.

## Supporting information

S1 Table Codon usage table for *Aurantiochytrium acetophilum*. (PDF)

S1 Raw images (PDF)

S2 Raw images (TIF)
