# Supplementary material for: Thraustochytrid hosts for expression of proteins relevant to SARS-CoV-2 intervention
Source: PLoS One. 2023 Apr 12;18(4):e0283592. doi: 10.1371/journal.pone.0283592 (PMC10096515; doi:10.1371/journal.pone.0283592)
Supplement: S1 Table — (PDF) [file pone.0283592.s001.pdf]

**Supplemental Table 1. Codon usage table for *Aurantiochytrium acetophilum***

| Codon | Amino Acid | Frequency | Codon | Amino Acid | Frequency | Codon | Amino Acid | Frequency |
|---|---|---|---|---|---|---|---|---|
| TAA | STOP | 8.2 | GGC | Gly | 30.3 | ATG | Met | 100.0 |
| TAG | STOP | 86.7 | GGG | Gly | 12.1 | AAC | Asn | 44.3 |
| TGA | STOP | 5.0 | GGT | Gly | 31.7 | AAT | Asn | 55.7 |
| GCA | Ala | 26.3 | CAC | His | 34.9 | CCA | Pro | 33.6 |
| GCC | Ala | 19.7 | CAT | His | 65.1 | CCC | Pro | 16.9 |
| GCG | Ala | 28.0 | ATA | Ile | 11.7 | CCG | Pro | 30.7 |
| GCT | Ala | 26.0 | ATC | Ile | 35.9 | CCT | Pro | 18.8 |
| TGC | Cys | 54.7 | ATT | Ile | 52.4 | CAA | Gln | 63.1 |
| TGT | Cys | 45.3 | AAA | Lys | 66.0 | CAG | Gln | 36.9 |
| GAC | Gln | 39.2 | AAG | Lys | 34.0 | AGA | Arg | 12.4 |
| GAT | Gln | 60.8 | CTA | Leu | 8.8 | AGG | Arg | 8.9 |
| GAA | Asp | 68.3 | CTC | Leu | 21.3 | CGA | Arg | 16.3 |
| GAG | Asp | 31.7 | CTG | Leu | 13.5 | CGC | Arg | 26.8 |
| TTC | Phe | 41.1 | CTT | Leu | 30.1 | CGG | Arg | 9.7 |
| TTT | Phe | 58.9 | TTA | Leu | 9.3 | CGT | Arg | 25.9 |
| GGA | Gly | 25.9 | TTG | Leu | 17.1 | AGC | Ser | 29.0 |

| Codon | Amino Acid | Frequency |
|---|---|---|
| AGT | Ser | 24.80 |
| TCA | Ser | 1.86 |
| TCC | Ser | 22.50 |
| TCG | Ser | 0.75 |
| TCT | Ser | 21.12 |
| ACA | Thr | 23.94 |
| ACC | Thr | 22.99 |
| ACG | Thr | 31.51 |
| ACT | Thr | 21.57 |
| GTA | Val | 18.77 |
| GTC | Val | 22.15 |
| GTG | Val | 26.79 |
| GTT | Val | 32.29 |
| TGG | Trp | 100.00 |
| TAC | Tyr | 36.48 |
| TAT | Tyr | 63.52 |
