# Supplementary figures and images for: Thraustochytrid hosts for expression of proteins relevant to SARS-CoV-2 intervention
Source: PLoS One. 2023 Apr 12;18(4):e0283592. doi: 10.1371/journal.pone.0283592 (PMC10096515; doi:10.1371/journal.pone.0283592)

Figure 3a – original blot

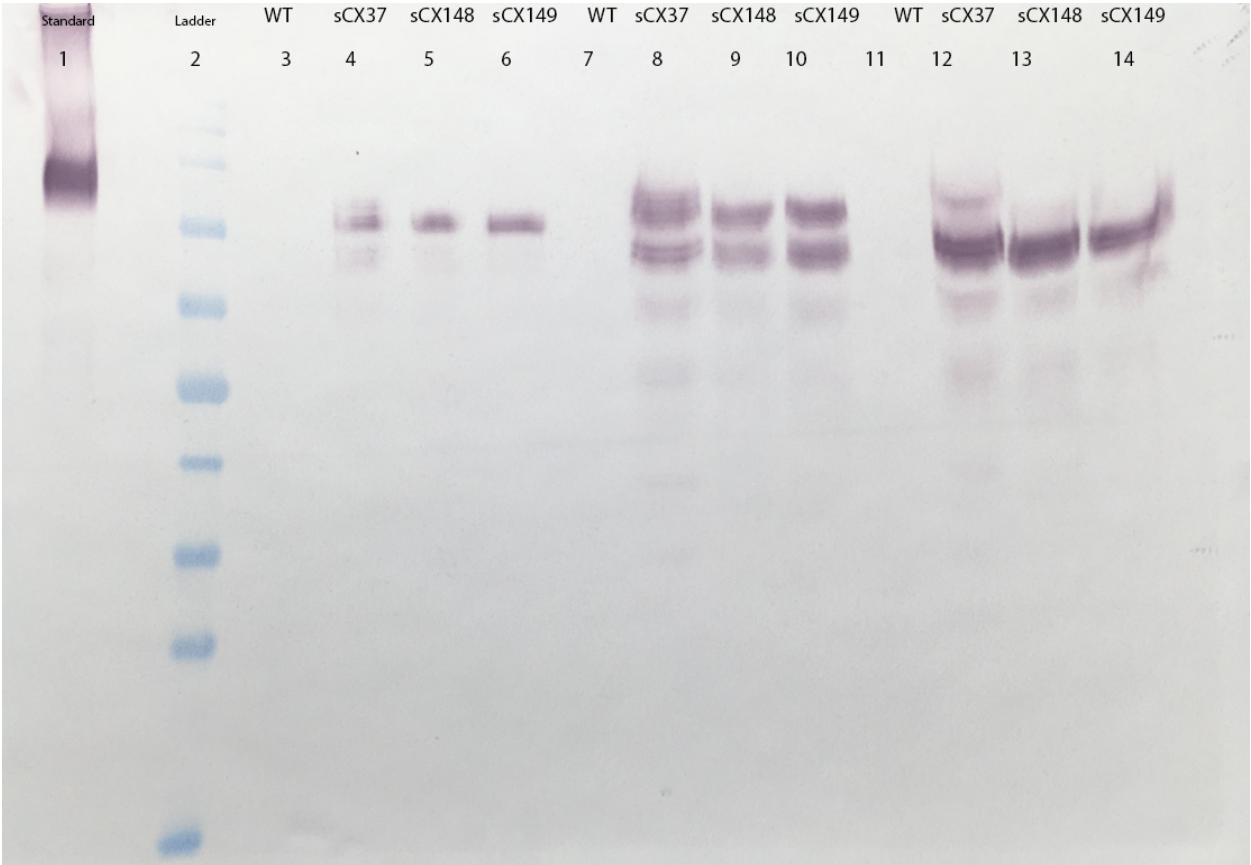

Loading Order Left to right (1-14)

Imaged captured with camera phone

Supplement: S1 Raw images — (PDF) [file pone.0283592.s002.pdf]

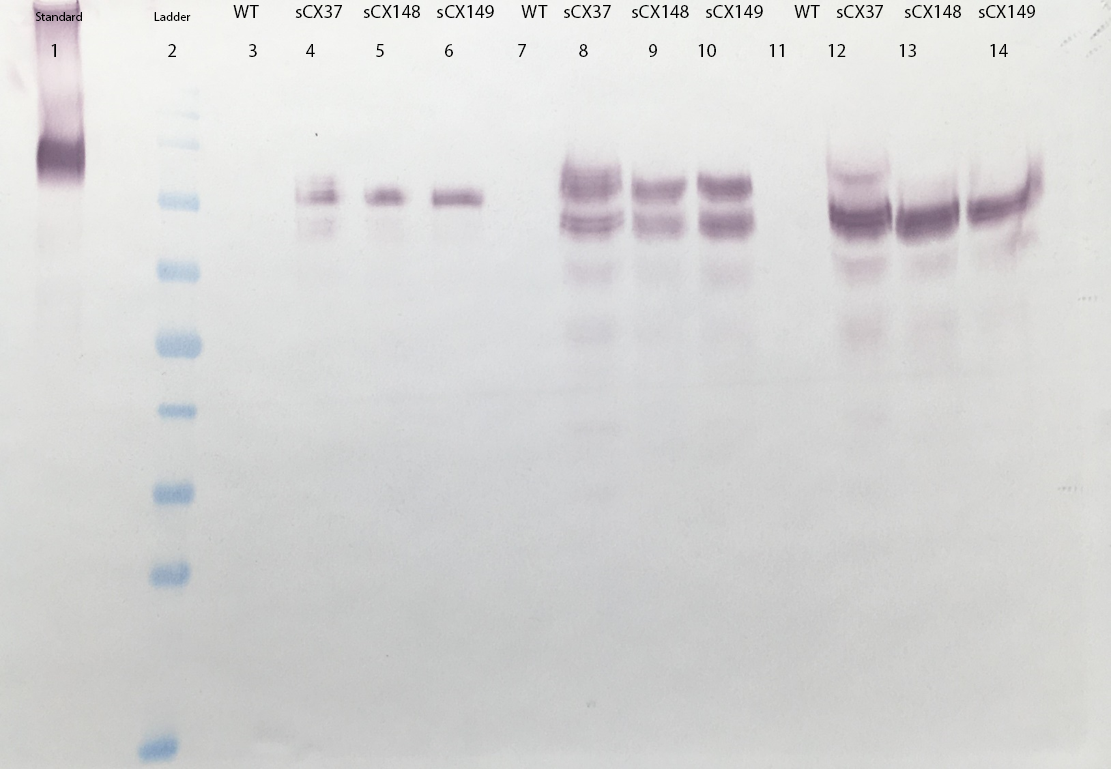

Supplement: S2 Raw images — (TIF) [file pone.0283592.s003.tif]
